# Archerfish foraging success varies with immediate competition level but not group size

**DOI:** 10.1093/beheco/arae040

**Published:** 2024-05-21

**Authors:** Dagmar der Weduwen, Nick A R Jones, Adèle Dubosque, Stefan Schuster, Keith T Sillar, Mike Webster, Luke Rendell

**Affiliations:** Centre for Biological Diversity, School of Biology, University of St Andrews, St Andrews, Fife, KY16 9TH, United Kingdom; Centre for Biological Diversity, School of Biology, University of St Andrews, St Andrews, Fife, KY16 9TH, United Kingdom; Centre for Biological Diversity, School of Biology, University of St Andrews, St Andrews, Fife, KY16 9TH, United Kingdom; Department of Animal Physiology, University of Bayreuth, NW I, Universitätsstr. 30, 95440 Bayreuth, Germany; Institute of Behavioural and Neural Sciences, School of Psychology and Neuroscience, University of St Andrews, St Andrews, Fife, KY16 9JP, United Kingdom; Centre for Biological Diversity, School of Biology, University of St Andrews, St Andrews, Fife, KY16 9TH, United Kingdom; Centre for Biological Diversity, School of Biology, University of St Andrews, St Andrews, Fife, KY16 9TH, United Kingdom

**Keywords:** boon, fish behavior, foraging, group behavior, kleptoparasitism, social behavior

## Abstract

Group living can lead to kleptoparasitism, the theft of resources by competitors. Under such conditions, foragers may alter their behavior to minimize competition. However, it is unclear how such behavioral changes impact foraging performance. Archerfish (*Toxotes* spp.) are a good model for investigating the behavioral responses to kleptoparasitism, as their hunting method (shooting waterjets at insects perched above the water) leaves them vulnerable to theft. They must hit the target prey with sufficient force to dislodge it; thus, the prey may land some distance away from the shooter. Kleptoparasitism rates increase with group size in archerfish, and individuals alter their behavior around conspecifics. We investigated whether group size affected shooting success, using 7-spot archerfish *T. chatareus*. We considered a fish’s shot to be successful if it knocked a fly, placed on a transparent platform above the tank, into the water. The probability of shooting success was modeled as a function of group size, aiming duration, nearest neighbor distance and position, and trial number. We found no effect of group size, aiming duration, or nearest neighbor distance or position on shooting success. Shooting success increased as trials progressed, likely due to the fish becoming more familiar with the task. We also found no change in the kleptoparasitism rate between group sizes. Instead, the likelihood of the shooter consuming the prey depended on the types of competition present at the time of shooting. We suggest that archerfish shooting behavior can be influenced by the presence of conspecifics in ways not previously considered.

## Teaser

If there is more competition around, you may try to be a bit more subtle when you try to feed. Archerfish spend less time aiming at their prey when in larger groups, although this does not change how likely they are to shoot the prey down. It also does not affect how likely they are to eat the prey, which is instead decided by how close and how fast other fish are moving before the shot.

## Introduction

The behavior of many animals is shaped by their social environment. Group living is seen across the animal kingdom, as it brings a variety of benefits, including protection from predators, faster food source discovery, and easier access to mates (Krause and Ruxton 2002; Barnard 2004; Ward and Webster 2016). However, group living also incurs costs, the largest typically being competition for resources. With the exception of socially cooperative species, the larger the group the faster the resources deplete (Hake and Ekman 1988; Thiebault et al. 2014), and the less food is available to each individual (Stenberg and Persson 2005). The mechanisms of such competition are varied. Scramble competition is often present, as individuals will race to get as much of the available resources before the food source is depleted. Competition can also take the form of kleptoparasitism, the active stealing of a resource from a competitor (Broom and Ruxton 2003), or aggressive contests, where individuals physically fight or intimidate competitors (Ryer and Olla 1995).

The effects of competition can also play out in more subtle ways. To avoid the costs of attracting competitors and kleptoparasites, foragers may need to pay attention to the distribution of the rivals, which in itself may be costly. Furthermore, the individual who initially discovers a patch has the advantage of gaining resources from that patch in the time between its initial discovery and the arrival of competitors (Giraldeau and Caraco 2000), while hunting foragers are attuned to cues from others and join those who are already foraging successfully (Webster et al. 2019). Those who have found food may thus be under pressure not to reveal that source to those around them and monitor conspecifics to determine whether they are at risk of being kleptoparasitized (Bugnyar and Heinrich 2005). In this way, competitors can interfere with an individual’s foraging efforts even in the absence of overt aggression as individuals have to be aware of the presence and proximity of rivals (Cresswell 1997). Kleptoparasitism can, therefore, have clear costs beyond loss of prey, being forced to spend less time with their prey, or increasing their foraging efforts to make up for the lost resources (Allen et al. 2021).

The risk of kleptoparasitism varies widely across and between species in response to several factors. Predators feeding on items requiring longer handling times tend to be at greater risk of having their food stolen (Steele and Hockey 1995), and less experienced or younger foragers may be at greater risk of being kleptoparasitized (Ridley and Child 2009). Juveniles may also show greater rates of kleptoparasitizing than adults (Steele and Hockey 1995), as food that has already been uncovered by another individual may be easier or less costly to access for less experienced foragers (Broom and Ruxton 2003). Theft of resources that would normally be out of reach is quite commonly seen, for example, gray reef sharks *Carcharhinus amblyrhynchos* kleptoparasitize whitetip reef sharks *Triaenodon obesus*, as the latter is capable of accessing prey in smaller crevices than the former (Labourgade et al. 2020). Such costs lead us to expect selection for behaviors that reduce the risk of kleptoparasitism.

Foraging individuals may minimize the risk of kleptoparasitism by altering their own behavior. For example, the distance between individuals may be increased or group size decreased to reduce the chance of interference or evasion tactics such as food caching deployed (Cresswell 1997). Evasion methods may also be deployed during food caching itself to prevent competitors from discovering the true caches (Bugnyar and Heinrich 2005; Leaver et al. 2007). However, it is unclear how such behavioral tactics affect foraging success, which is important to understand the trade-offs involved at the individual level. Archerfish (*Toxotes* spp.) are a good model for investigating the behavioral responses to the threat of kleptoparasitism. These fish prey on insects above the water’s surface, which they shoot down by spitting a concentrated jet of water at the target (Gill 1909). The shooter is left open to kleptoparasitism, although it does not as yet physically possess the prey, as another individual may reach the dislodged food item first (Rischawy et al. 2015). Archerfish evolution has co-opted an escape mechanism found in many fish, called a C-start, to quickly reach falling prey. The fish bends its body into a C-shape to rapidly change direction and accelerate toward the prey, using the prey’s falling trajectory to calculate the speed required to reach the prey at the moment it impacts the water (Reinel and Schuster 2014). Kleptoparasitism is common in 7-spot archerfish (*T. chatareus*), with loss rates for shooters reported in one lab-based study to be around 44% (Dill and Davis 2012). This study also reported that the rate of kleptoparasitism increases with group size from 3 to 5 individuals but does not increase further in larger groups (Dill and Davis 2012). Archerfish alter their shooting behavior in the presence of a single conspecific, with fish taking longer to shoot overall, making more orientations while aiming, and being closer to the target when they do shoot (Jones et al. 2018). This combination of kleptoparasitism and sensitivity to social environment makes archerfish a good model in which to study how the threat of kleptoparasitism, represented as group size, influences shooting behavior.

We used 7-spot archerfish to investigate whether changes in a shooter’s behavior in response to the threat of kleptoparasitism affect their foraging success. Because of the manner in which archerfish hunt, we were able to separate overall foraging success into 2 different stages: success in shooting the prey down into the water and success in consuming the prey. We will refer to these 2 components as “shooting success” and “intake success,” respectively, throughout this article.

Here, we set out to determine whether shooting success—the ability to knock a prey item off a platform and into the water—is affected by group size due to the potential changes in kleptoparasitism threat represented by the varying numbers of competitors. We expected shooting success to be greater in smaller groups, due to the decreased competition (Dill and Davis 2012), and that this relationship may be influenced by aiming duration, assuming longer aiming times result in greater accuracy. We also expected nearest neighbor distance and position to affect aiming duration as individuals are sensitive to and adjust their aiming when a conspecific is visible (Jones et al. 2018) and therefore predicted that success would be greater when nearest neighbors were further away or facing away from the shooter.

We also investigated whether the shooter’s intake success changed in relation to group size, and whether it was affected by the behavior of their neighbors. We used 2 measures of kleptoparasitism threat, proximity to the shooter when it takes a shot (≤1 body length away), and other fish C-starting toward the predicted landing spot as the prey falls, and analyzed how each type affected prey consumption by the shooter. We predicted that the shooter’s intake success would be higher in groups of 3 than in groups of 5, and the intake success would be lowest if both types of competition were present.

## Methods

### Subjects and husbandry

We used 60 seven-spot archerfish, *T. chatareus*, in the experiment. Fish ranged from 8 to 15 cm in length. As archerfish are sexually monomorphic, we are unsure of the sex ratio of the groups used in this experiment. Groups of 3 or 5 were formed by size-matching fish, keeping fish in experimental groups within 1 cm of each other in length, and each individual group was formed from the same stock tank to ensure familiarity and thereby reduce the likelihood of aggression. The fish had not been previously exposed to experimental conditions.

The study was conducted in the fish laboratory in the Department of Animal Physiology at the University of Bayreuth, Germany. The fish were housed in 7 identical-sized (120 × 60 × 60 cm) stock tanks in the same room. Temperature and water conditions were matched across all tanks. The water was brackish, maintained at a conductivity of 3.5 to 3.7 mS cm^−1^, and nitrates and nitrites were kept low. 30% water changes were conducted every 2 wk. Each tank had a layer of gravel for enrichment and was equipped with 2 Eheim internal aquaball filters. The room temperature was maintained between 26 and 27 °C with a light cycle of 12/12 h light/dark. Water temperature was controlled primarily by room temperature, but each tank also contained a large submersible thermostat-controlled heater (450 W). Fish were fed pellet food (Sera Cichlid Sticks) daily.

The behavioral trials run in this study were approved by the University of Bayreuth. The procedures used in this experiment were also in accordance with the ethical standards of the University of St. Andrews. No fish died or suffered ill health during this study, and all individuals were retained in the laboratory for future use. None of the procedures used in this study required UK Home Office licensing. All tanks were enriched with plastic plants for cover, and handling was kept to a minimum. When fish were moved between tanks, they were caught using 2 large hand nets to reduce the likelihood of extended capture periods. During our study, we closely monitored each fish, specifically for signs of reduced feeding rate, responsiveness, stereotypic behavior, and color changes. We observed very few instances of these signs, and they were only temporary and only occurred in the period immediately after fish had been transferred between tanks.

### Experimental setup

**Fig. 1.**
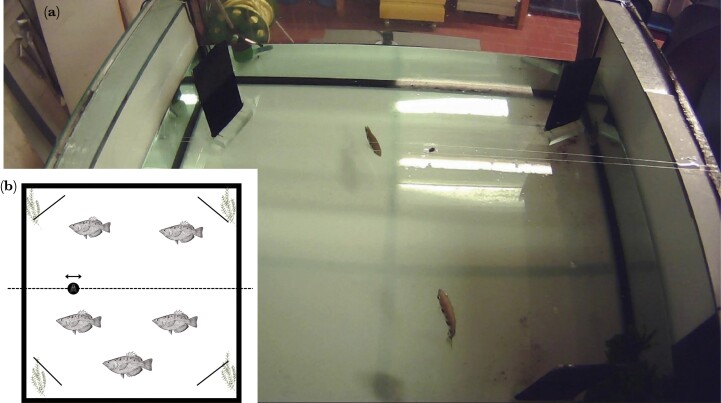
(a) View of experimental tank with a fly presented above a group of 3 fish during a trial. (b) Diagram showing the tank layout from above.

We placed each group of 3 or 5 fish in one of the 2 identically set-up tanks of 150 × 150 × 50 cm (Fig. 1).

Each tank had a bare floor with a white base to ensure that the fish were visible for an overhead camera used to record each session. Environmental enrichment was provided in the form of 4 large plastic plants of equal dimensions (40 cm high broad-leafed bush replica with ceramic base) placed in 2 corners and 4 black opaque screens, one in each corner. Each tank also contained 2 Eheim internal aquaball filters and 2 large submersible heaters. Fish were moved into the experimental tanks between 16:30 and 17:00 and left to acclimate for approximately 40 (39.5–41) h before testing sessions started. Each group experienced 2 experimental sessions per day starting at approximately 9:30 and 16:30, respectively.

A conveyor system was suspended over each tank to allow the food items to be moved into position above the tank while minimizing disturbance. This conveyor was constructed out of a small transparent square plastic platform, thus allowing the fish to see the food, mounted onto 2 monofilament lines that allowed the platform to move along the conveyor. The platform was 25 cm above water level such that the fish were more likely to shoot than jump at the food (Shih et al. 2017).

We tested 10 groups of 3 and 10 groups of 5 during this experiment. A minimum of 10 experimental sessions were conducted with each group. More sessions were conducted if the fish were unresponsive, defined as when a group made 2 shots or fewer in the whole session, during one or more of the initial sessions until 10 sessions were conducted with at least 1 shot being made in at least 8 out of 10 trials per session. Each session consisted of multiple trials, normally 10 trials (range 8 to 12 depending on conditions specified below). Each trial started when a thawed fly (frozen house fly, *Calliphora* sp.) was suspended above the tank on the conveyor platform. The fly would remain suspended until it was knocked off by an archerfish’s shot, knocked off by a jumping archerfish (although this was a rare occurrence, at <0.1% of all trials), or fell off the platform due to manipulation of the conveyer by the experimenter (<0.5% of all trials). A trial ended when a fly had been knocked off the platform and a session ended after 10 trials in which the fly was knocked off by a shot. Additional trials were run if a previous trial had ended due to a fly falling without being shot. Each session was recorded using the overhead camera (ELP 5 Megapixel USB webcam recording 30fps) connected to a laptop running Debut Video Capture software.

### Data analysis

We used Solomon Coder software (https://solomon.andraspeter.com/), to view the videos at a speed of one frame every 0.2 s (thus viewing 1 in 6 frames). Each fish was identified by its markings and size in relation to the other fish present in the experimental tank and given a number. For every shooting event that occurred, we recorded the identity (number) of the shooter and nearest neighbor, whether the nearest neighbor was facing toward (the shooter within a 90° field of view of the nearest neighbor) or away from the shooter, and the distance (in body lengths) between the 2 fish. We also recorded the time (since the start of the trial) at which the shot occurred, whether the shot knocked the food off the platform, and the time the shooter took to aim at the food before shooting. Aiming behavior was evident from the orientation of the archerfish, as they tilt backward to line up the shot in the vertical plane (Dill 1977), and the aiming duration ended when the shot was released. After every successful shot, we identified what indices of kleptoparasitism threat were present. There were 4 options: at least one fish being within one body length of the shooter, a fish other than the shooter C-starting toward the falling prey, both types of competition present, or no competition. We then identified which fish ate the prey, either the shooter, the nearest neighbor, another individual, or we noted that it was unclear which occurred sometimes when multiple fish reached the prey at the same time. We also made note of the group size and the trial number for each shot. Trial number was continuous across sessions within each group, and trial numbers above 157 were excluded as there was only one group that reached each of those high trial numbers, which resulted in this group having nearly double the amount of data as the other groups, thereby skewing the data. Fish used in trials of groups of 3 were sometimes reused in trials of groups of 5, but new identity numbers were assigned within each group. Videos of 3 groups, one group of 3 and 2 groups of 5, were deemed unreliable as the lighting conditions or camera angle made identification of the individual fish difficult, and thus, 31 out of 200 (15.5%) videos were not coded or included in the analysis. The data were coded by 2 separate people, and we calculated Krippendorf’s α reliability coefficient to determine how consistent the coding was between them (α = 0.89).

The unit of analysis was single shots by individual fish. We combined the data from each video and assigned group and session identity to each single shot, which was coded 0 if the shot failed to dislodge the prey and 1 if the shot did. Data points for which the nearest neighbor information was unavailable were removed from the dataset (7% of the total). Initial exploration determined that the nearest neighbor position and distance variables were confounded, as fish were more likely to be facing toward the shooter when they were closer. To prevent these confounding effects from unjustly influencing our analysis, nearest neighbor distance and position were grouped into one variable with 4 levels: ≤1 body length away and facing away from the shooter; ≤1 body length away and facing toward the shooter; ≥2 body lengths away and facing away from the shooter; and ≥2 body lengths away and facing toward the shooter. This allowed us to test whether orientation or distance was most influential in affecting shot success.

We conducted statistical analysis in R, version 4.0.2. We constructed binomial family generalized linear mixed models using the *lme4* package (Bates et al. 2015) to fit the probability of a shot being successful as a function of group size. All model assumptions were tested using residual diagnostic plots in the DHARMa package in R (Hartig 2019). We included additional predictors in the model attempting to mitigate potential confounding effects of aiming duration, nearest neighbor distance and position, and trial number. Group size was a factor with levels “3” and “5” corresponding to the number of fish. Aiming duration was the total time in seconds that the shooter spent aiming at the target before shooting. This was indicated by the archerfish orienting itself at an angle near or below the food, its head facing upwards. We also included trial number (counted across all trials for that group). We had 2 opposing but plausible predictions for the effect of trial number on shooting success. Either the archerfish would become satiated throughout the experiment and shooting success would decrease with trial number, or the archerfish would become more familiar with, and focused on, the food delivery mechanism, and shooting success would increase with trial number. Thus, experience during the experiment could potentially increase or decrease success, but in whichever case we wanted to incorporate that effect in our modeling. Group and session identity were included as intercept-only random effects since groups could have had different baseline success (because of differences in the individuals they contain), and sessions could be subject to temporary effects (e.g. varying noise levels on different days), with session ID nested within group ID. We tested models that did not include shooter identity as a random effect, and while there appeared to be an effect of nearest neighbor distance and position on shooting success in these models, this effect was attributed entirely to a single fish that favored shooting when the nearest neighbor was more than 2 body lengths away and facing away. We therefore decided to include shooter identity as a random effect to control for the variation attributed to individual fish, nested within group ID. The final model was thus written in R/*lme4* syntax as glmer(Success ~ Group Size + Aiming Duration + NN Dis. Pos. + Trial Number + (1|GroupID: ShooterID) + (1|GroupID: SessionID), family = binomial).

On obtaining the estimates of the model testing our main experimental question, we constructed 2 additional models to explore other aspects of the data we had collected. Firstly, we wanted to examine whether group size affected shooting behavior without influencing shooting success, as shooting is a costly behavior, and the shooter may change their behavior in response to group size in such a way that it does not influence success alone but also, for example, the latency to shoot. Secondly, we wanted to explore the idea that aiming duration might act as public information, predicting that if so, durations should be reduced in larger group sizes as the risk of detection is higher with more observers. The first model thus estimated the effects of group size and the nearest neighbor’s distance and position on aiming duration, assuming Gaussian errors after plotting the residuals: glmer(Aiming Duration ~ Group Size + NN Dis. Pos. + (1|GroupID: SessionID)). Group and session ID were included as random effects, with session ID nested within group ID, but shooter ID was removed as a random effect from this model as there was <0.0005 variance attributed to it in a model that initially included it. Furthermore, we wanted to investigate whether the number of shots per trial changed with group size, as the act of shooting is also likely to act as public information, leading us to expect fewer shots per trial in larger groups. The second model, therefore, predicted the total number of shots in a trial as a function of group size and trial number while including group ID, session ID, and shooter ID as random effects, with session and shooter ID nested within group ID, and assuming Poisson errors for count data: glmer(Number of Shots ~ Group Size + Trial Number + (1|GroupID: ShooterID) + (1|GroupID: SessionID),family = poisson). The “Number of Shots” variable was scaled using the scale() function in R, as without scaling the model produced a very large eigenvalue.

To investigate the likelihood of the shooter consuming the prey (i.e. not being subject to kleptoparasitism), we constructed a separate model on a subset of the data. We removed all data points where shooting success was 0 and removed any data points where it was unclear which individual consumed the prey. The resulting dataset contained 1,244 observations, representing 66% of the successful shooting events. We fitted a binomial model to a 1/0 response variable, which took the value 1 when the shooter obtaining the prey and 0 otherwise. Model predictors were group size and the level of competition present (no competition, nearest neighbor within 1 body length of the shooter, other individual c-starting toward the prey, both types of competition present), with group identity as a random effect. Although we initially also included session identity and the identity of the coder as random effects, the variance assigned to these variables was less than 0.0005, and therefore, they were removed from the model. The fitted model was therefore coded in R as glmer(Consumer ~ Group Size + Competition + (1|Group ID), family = binomial). As a follow-up, we conducted a post hoc GLMM to determine whether the frequency of each competition level per session of 10 shots varied with group size and competition level. The data followed a Poisson distribution, and the fitted model was therefore coded in R as glmer(Frequency ~ Group Size × Competition + (1| Group ID), family = Poisson).

Predicted *R*^2^ values were estimated for each model using the MuMIn package (Barton 2009). All models were checked for collinearity by calculating the variance inflation factors using the performance package (Lüdecke et al. 2021), and we found low collinearity between all variables in each model, which did not contain interaction terms. Predicted mean probabilities and associated confidence intervals for shooting success and intake success were obtained for each model using the ggeffects package (Lüdecke 2018). Figures were constructed using ggplot2 (Wickham 2016).

## Results

A total of 3,082 shooting events were analyzed, occurring across 175 sessions with 17 groups. A total of 70 shooters were recorded across all sessions. Shots tended to be successful (1,870 successful vs. 1,212 not successful), and there were more shots in the groups of 5 than in the groups of 3 (1,842 shots vs. 1,239 shots), although there was a similar mean number of shots per shooter in both groups (46 and 43 shots per fish in groups of 3 and 5, respectively).

Shooting success was not affected by group size in our experiment (Table 1).

**Table 1.** General(ized) linear mixed model results. (A) glmm results for the model testing the main experimental question (*R*^2^ = 0.460), (B) glmm results for the first post hoc analysis (*R*^2^ = 0.186), and (C) glmm results for the second post hoc analysis (*R*^2^ = 0.269). (D) glmm results for the model testing the likelihood of the shooter consuming the prey (*R*^2^ = 0.400), and (E) glmm results for the third post hoc analysis (*R*^2^ = 0.277). Significant (*P *< 0.05) estimates are shown in bold.

A: Shooting success modeled as a function of group size, aiming duration, nearest neighbor distance and position, and trial number (a priori hypothesis).	
Fixed terms	Coefficient ± SE
Intercept	0.153 ± 0.388
Group size = 5	0.466 ± 0.446
Aiming duration	0.006 ± 0.065
Nearest neighbor distance and position = 1BL, facing toward	0.015 ± 0.159
Nearest neighbor distance and position = 2 + BL, facing away	−0.295 ± 0.187
Nearest neighbor distance and position = 2 + BL, facing toward	−0.148 ± 0.191
Trial number	**0.005 ± 0.002**
Random terms	Variance ± SD
Group identity: shooter identity	2.594 ± 1.612
Group identity: session identity	0.259 ± 0.509
B: Aiming duration modeled as a function of group size and nearest neighbor distance and position (post hoc hypothesis).	
Fixed terms	Coefficient ± SE
Intercept	**0.574 ± 0.049**
Group size = 5	**−0.347 ± 0.043**
Nearest neighbor distance and position = 1BL, facing toward	0.019 ± 0.044
Nearest neighbor distance and position = 2 + BL, facing away	0.082 ± 0.050
Nearest neighbor distance and position = 2 + BL, facing toward	−0.028 ± 0.053
Random terms	Variance ± SD
Group identity: session identity	0.040 ± 0.199
Residual	0.535 ± 0.731
C: Total number of shots per trial modeled as a function of group size and trial number (post hoc hypothesis).	
Fixed terms	Coefficient ± SE
Intercept	0.258 ± 0.156
Group size = 5	0.065 ± 0.249
Trial number	**−0.001 ± 0.0007**
Random terms	Variance ± SD
Group identity: shooter identity	0.440 ± 0.664
Group identity: session identity	0.065 ± 0.256
Residual	0.680 ± 0.825
D: Likelihood of the shooter consuming the prey modeled as a function of group size and competition (a priori hypothesis).	
Fixed terms	Coefficient ± SE
Intercept	**3.861 ± 0.511**
Group size = 5	−0.103 ± 0.354
Competition = 1 body length away	**−1.961 ± 0.641**
Competition = C-start	**−2.868 ± 0.469**
Competition = both	**−3.457 ± 0.468**
Random terms	Variance ± SD
Group ID	0.405 ± 0.637
E: Frequency of competition levels per session modeled as a function of group size and competition level (post hoc hypothesis)	
Fixed terms	Coefficient ± SE
Intercept	**0.962 ± 0.097**
Group size = 5	0.161 ± 0.151
Competition = 1 body length away	−0.412 ± 0.211
Competition = C-start	0.185 ± 0.107
Competition = both	0.073 ± 0.111
Group size = 5: Competition = 1 body length away	−0.446 ± 0.315
Group size = 5: Competition = C-start	**−0.343 ± 0.168**
Group size = 5: Competition = both	**0.418 ± 0.161**
Random terms	Coefficient ± SE
ID	0.021 ± 0.144

**Fig. 2.**
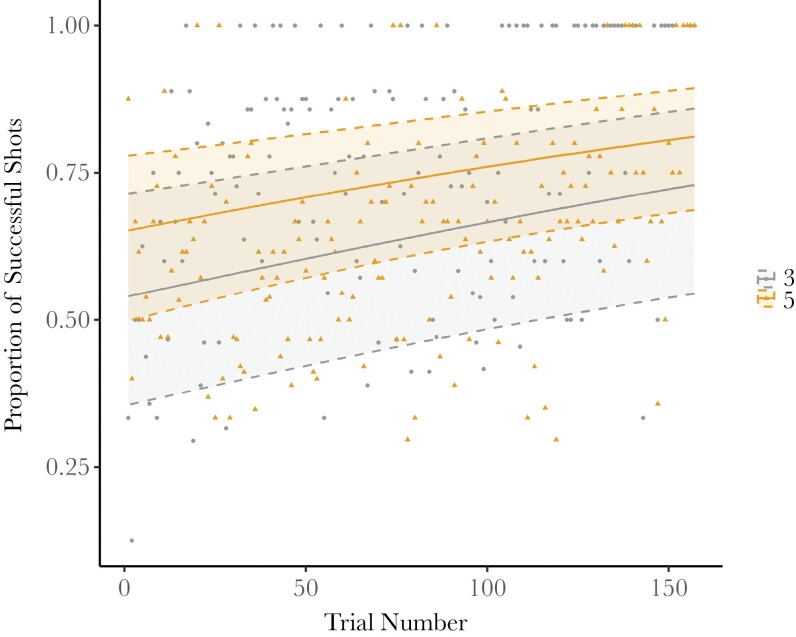
Proportion of successful shots per group per trial. The proportion of successful shots increases at later trials for groups of both sizes. The points represent the raw data (gray circles for groups of 3 and yellow triangles for groups of 5). Shaded regions show the 95% confidence intervals.

When including random effects for group, session, and shooter identity, there was no statistically significant change in shooting success between groups of 3 or 5. The success of archerfish shooting did improve within sessions, increasing in later trials (Table 1, Fig. 2).

**Fig. 3.**
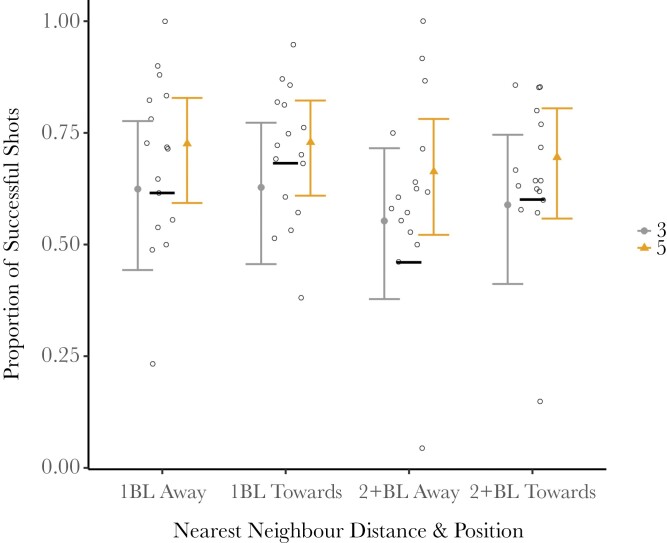
Proportion of successful shots per group size per nearest neighbor distance and position. “BL” stands for “body length” and “Away” and “Toward” refers to which way the nearest neighbor was facing in relation to the shooter. There is no difference in the shooting success with changes in the nearest neighbor’s distance and position. The black bars indicate mean proportions across all groups.

There were also no statistically significant effects of aiming duration and nearest neighbor distance and position on the success of archerfish shooting, and effect estimates were very small. Although there are multiple data points indicating low success when nearest neighbors are more than 2 body lengths away and facing away, these points come from a single fish that shot very frequently, and we thus could detect no overall average effect of nearest neighbor distance and position on shooting success (Fig. 3).

**Fig. 4.**
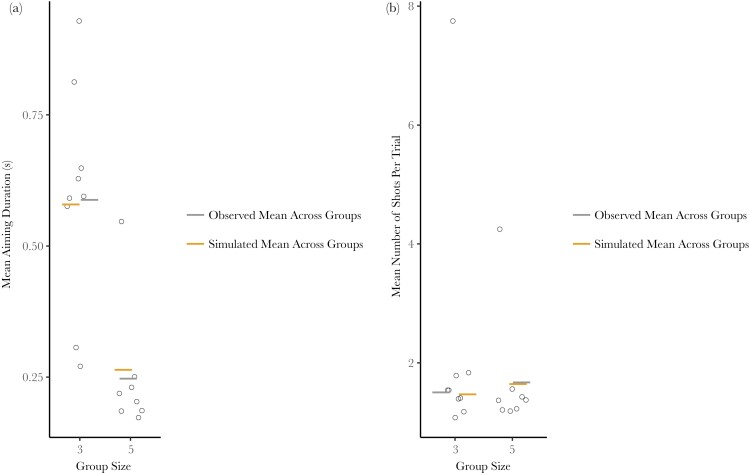
(a) Mean aiming duration in seconds in relation to group size. Open circles represent group means; bars represent means across all groups. The mean aiming duration is 0.39 s longer in groups of 3 than in groups of 5. This relationship remains the same even when the large outlier in group size 3 is excluded. (b) The mean number of shots per trial in relation to group size. Open circles represent group means; bars represent means across all groups. There are no significant differences in the mean number of shots per trial for different group sizes.

As we did not find the expected effects of group size and aiming duration on shooting success, we fitted 2 post hoc exploratory models to look for evidence of any possible underlying effect of group size on shooting behavior. When we included group and session identity as random effects, aiming duration was predicted to decrease with group size (Fig. 4A).

Shooter identity was not included as a random effect in this model as the proportion of variation attributed was negligible (<0.0005). In a second exploratory model, we found no effect of group size or trial number on the scaled number of shots taken during a trial (Fig. 4B) when including the group, session, and shooter identity as random effects.

**Fig. 5.**
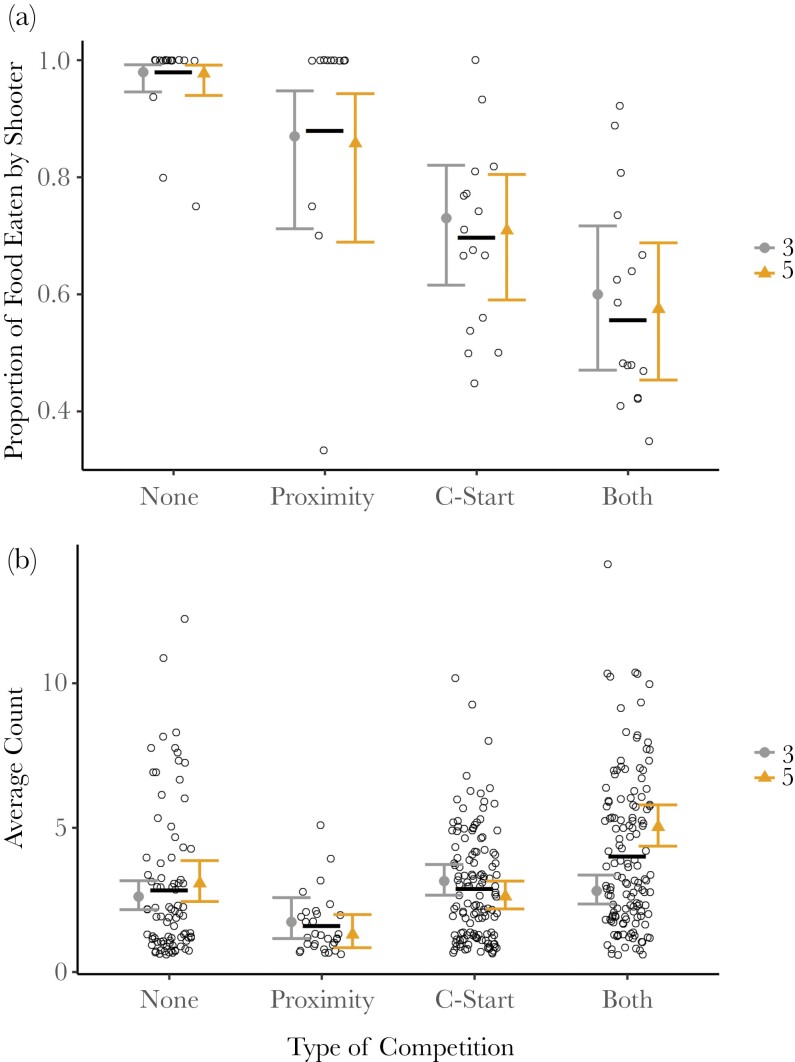
(a) The mean proportion of prey eaten by the shooter in relation to the type of competition present. The black bars indicate mean proportions across all groups. The likelihood of the shooter consuming the food decreases when another fish is within 1 body length of the shooter (proximity), when another individual C-starts toward the prey, or when both types of competition are present. There are no significant differences in the proportion of food eaten by the shooter for different group sizes. (b) Average count per session of each competition type per group size. The black bars indicate mean proportions across all groups. C-starting when more than one body length away is more common in groups of 3, and C-starting when within one body length is more common in groups of 5.

Finally, we found that there was a reduced likelihood of the shooter consuming the prey when competition was present, but this varied depending on the type of competition present (Table 1, Fig. 5A). When both another individual was within one body length of the shooter and another individual C-started toward the shooter, the probability of the shooter consuming the food decreased by approximately 40%. We also found that there was an increased frequency of competitors C-starting toward the prey from one body length away in groups of 5 and an increased frequency of competitors C-starting toward the prey from more than one body length away in groups of 3 (Table 1, Fig. 5B).

## Discussion

Foraging success was not affected by group size in our experiment. Shooting success was not affected by aiming duration or the behavior of the nearest neighbor, but the probability of successful shots increased with experience and exposure to the targets. We suggest that, as each session progressed, the fish became more familiar with the food delivery mechanism, thus leading to increased shooting success. The platform needed to be hit from the right angle and with the right amount of force to dislodge the food, so the task required some skill. Practice may have been required for the fish to adjust their shots to the right speed and angle, as they are known to improve their shooting abilities over time when faced with a new task or delivery mechanism (Schuster et al. 2006). However, despite our findings, we cannot rule out that speed-accuracy trade-offs may exist when greater precision is required (Jones et al. 2020). The target height in our experiment was relatively low given the typical shooting range for archerfish (Luling 1963), and if a higher target were to be presented, it is possible that aiming duration may impact shooting success.

In the wild, the presence of conspecifics is often a good indicator of the presence of food or other beneficial resources. Therefore, individuals often tend to investigate areas where conspecifics are present (Anderson 1991; Midford et al. 2000). A study in juvenile walleye pollock (*Gadus chalcogrammus*) determined that this type of local enhancement was only present when food was provided in clumps, and not when food was dispersed (Ryer and Olla 1995). This same study also found that when food was dispersed, some fish aggressively defended areas of their tank to prevent conspecifics from obtaining resources. We observed that some dominant shooters would monopolize the area near the target and chase away encroaching individuals. It is possible, therefore, that we did not observe group size affecting shooting success in regard to the perceived threat of kleptoparasitism because the other individuals were excluded by an aggressive dominant fish, i.e. that dominant shooters could reduce the risk of kleptoparasitism by the threat of aggression. It is conceivable that individual differences between shooters masked any changes made in shooting behavior in relation to group size. We should also note that the movements of the fish in our experimental tanks are likely to be constricted compared with natural conditions, although we cannot say if or how this may have influenced our results, as there is little research published on archerfish in the wild.

We were surprised not to find an effect of either group size or nearest neighbor distance and position on shooting success; however, this may be explained by our results on the shooter’s intake success depending on group size and competition. Dill and Davis (2012) established that the risk of kleptoparasitism to the shooter increases with a group size from 3 fish to 5 fish; thus we had expected to see changes in the shooters’ behavior to minimize the possibility that the food would be stolen. We further expected these changes in shooting behavior to influence the success rate, as we had predicted a reduced aiming duration in larger groups and, intuitively, that less time spent aiming would negatively impact shooting success. Our post hoc analyses do suggest that group size does affect the time shooters spend aiming, but, counter-intuitively, that this reduction in aiming duration in larger groups does not, in turn, influence shooting success. We also found no effect of group size on the shooter’s intake success, in contrast to the findings of Dill and Davis (2012). Their study determined that the rate of intraspecific kleptoparasitism increases with group size from 3 to 5 individuals but does not increase further at even greater group sizes. In contrast, we found that the risk of kleptoparasitism was mediated by the level of perceived kleptoparasitism threat present. Although there was a reduced likelihood of the shooter eating the food if another individual was within one body length at the time of shooting, the likelihood was not as low as when an individual more than one body length away C-started toward the prey (88% vs. 70%). Although this level of competition was more likely to occur in groups of 3, there was no difference in the likelihood of the shooter consuming the prey between groups overall. Therefore, the mere presence of a conspecific close to the shooter is not necessarily a large enough threat to alter the shooter’s behavior, and in our experiment, the fish were close enough together even in smaller groups that there was no difference in kleptoparasitism risk with group size. It is possible that our findings differed from those of Dill and Davis (2012) as our model included the different types of competition present, which is itself affected by group size. Although we did not find increased levels of kleptoparasitism at increased group size, competition is more likely, and we found that kleptoparasitism rates differ with different types of competition. Therefore, it is possible that the competition types present is the underlying cause of Dill and Davis (2012) findings.

If archerfish success in shooting down a target is not linked to the time spent aiming, why would fish in smaller groups increase their aiming duration? The difference in aiming duration may seem small, but archerfish hunting sequences happen incredibly fast; previous findings report decision making during hunting to occur on the scale of milliseconds (Schlegel and Schuster 2008). Thus, for an archerfish, 0.39 of a second could be a serious delay. Although we considered that it is possible that the angle from which the target is shot may impact the shooter’s likelihood to reach the downed food, our findings in this study appear to not support this theory. It is still possible that increased aiming duration makes it more likely for conspecifics to notice the behavior and become aware of the prey item; however, we must consider other theories as to why aiming duration is longer in smaller groups. One possibility is that, given shooting water is conspicuous outside of the water (Schuster 2018), the increased aiming duration is an anti-predator response. In smaller groups, the risk of predation is greater; therefore, increased time in the aiming position may allow the shooter to scan for predators for longer. Further research is required, including examining how likely the shooter is to get the reward as a function of its own position relative to the target, to determine whether the changes in aiming duration are an anti-predator response, a counter-kleptoparasitism response, or a combination of the 2.

Overall, we found little evidence of adjustments in archerfish behavior in response to perceived kleptoparasitism risk with increasing group sizes. Our results, however, suggest some evidence that archerfish shooting accuracy increases as trials progressed. We found no evidence that archerfish aiming duration affects shooting success, but some limited evidence that aiming duration does decrease with group size. We also found that the shooter’s intake success depends on the level of perceived kleptoparasitism threat but not group size. Our findings suggest that the interaction between effects like public information use and kleptoparasitism defence are perhaps more complex than we initially thought.

## Data Availability

The analysis reported in this article can be reproduced using the data provided by der Weduwen et al. (2024).
